# Acoustic one-way metasurfaces: Asymmetric Phase Modulation of Sound by Subwavelength Layer

**DOI:** 10.1038/srep28023

**Published:** 2016-06-16

**Authors:** Xue Jiang, Bin Liang, Xin-ye Zou, Jing Yang, Lei-lei Yin, Jun Yang, Jian-chun Cheng

**Affiliations:** 1 Key Laboratory of Modern Acoustics, MOE, Institute of Acoustics, Department of Physics, Nanjing University, Nanjing 210093, P. R. China; 2 Collaborative Innovation Center of Advanced Microstructures, Nanjing University, Nanjing 210093, P. R. China; 3 State Key Laboratory of Acoustics, Chinese Academy of Sciences, Beijing 100190, P. R. China; 4 Imaging Technology Group, Beckman Institute, University of Illinois at Urbana-Champaign, Urbana, Illinois 61801, USA; 5 Key Laboratory of Noise and Vibration Research, Institute of Acoustics, Chinese Academy of Sciences, Beijing 100190, P. R. China

## Abstract

We theoretically design and numerically demonstrate an acoustic one-way metasurface, which is a planar and acoustically subwavelength layer behaving like a nearly-reflectionless surface with arbitrary wave-steering capability for incident wave impinging on one side, while virtually blocking the reversed wave. The underlying mechanism is based on an asymmetric phase modulation by coupling a phase array and a near-zero-index medium. We exemplify a metastructure-based implementation by combining the hybrid metastuctures and labyrinthine structures. Moreover, the performance of the proposed implementation is demonstrated via three distinct phenomena of anomalous refraction, wave splitting and conversion of propagation wave to surface wave. Our findings may offer more possibilities for sound manipulation and improve the application potential of acoustic artificial devices in situations such as ultrasonic imaging and therapy.

The recent emergence of acoustic metamaterials provides acoustic properties unavailable in nature^1^,^2^,^3^,^4^,^5^,^6^,^7^,^8^,^9^,^10^, and has enabled design of various conceptual devices with special wave-manipulation functionalities such as invisibility and cloaking^2^,^3^, sub-diffraction imaging lens^5^,^6^, acoustic field rotator^7^, etc. In particular, acoustic metasurfaces^10^,^11^,^12^,^13^,^14^,^15^,^16^,^17^,^18^ have aroused increasing interests due to the ability to control the transmitted and reflected phase of waves to generate diverse functionalities with a planar layer of subwavelength thickness. The existing designs of metasurfaces, however, only yield completely symmetric manipulations for incident wave impinging on the two opposite sides of the metasurfaces, which poses a fundamental challenge for the development of wave-steering devices. The possibility of breaking through the barrier of how to realize asymmetric acoustic transmission has attracted extensive interests^19^,^20^,^21^,^22^,^23^,^24^,^25^,^26^,^27^,^28^ and is believed to have the potential to improve the quality of medical ultrasound imaging^20^,^27^. Despite the recent progresses in the acoustic one-way devices developed in both linear^24^,^25^,^26^,^27^,^28^ and nonlinear regimes^19^,^20^,^21^,^22^,^23^, the resulting “acoustic diodes”, which generally have a bulky size, are only designed to possess asymmetric transmission of energy flux but unable to provide free manipulation on the transmitted wavefront via rational design of their structural parameters. The great potential of combining the capability of one-way manipulation and the flexibility of wavefront steering have not yet been explored, which should have deep implications for acoustic devices, acoustic-based applications and the field of acoustics in general.

In this article, we theoretically design and numerically realize an acoustic one-way metasurface (AOM) capable of freely tailoring the transmitted wave for incident wave impinging on one side but prohibiting the reversed wave from passing. Unlike the previous metasurface schemes giving rise to identical phase profile for incident wave from two sides, on which a versatile but unavoidably symmetric sound manipulation is based, the current AOM design has an inherently different mechanism that generates an asymmetric phase modulation along two opposite directions. Such a unique ability is realized by using a combination of acoustic phase array (PA) with the flexibility of free phase-steering and near-zero-index material (ZIM) with the important feature of high phase selectivity and phase preservation. We have also demonstrated a practical implementation of our design by artificial structures, in which a hybrid metastucture^16^ with full phase control is employed to freely steer the transmitted wave^16^ and a labyrinthine structure^27^,^29^ is used to effectively mimic a ZIM^30^. Performances of the proposed AOM are demonstrated by realizing three distinctive phenomena of anomalous refraction, splitting acoustic wave and converting the propagating wave into surface wave. Combining the capability of asymmetric transmission and flexibility of manipulating the wave profiles together, the realization of AOM allows for more versatile manipulation of sound and opens up possibilities for the design of asymmetric manipulation devices that may find applications in various situations such as ultrasonic imaging and therapy.

## Results

**Figure 1:**
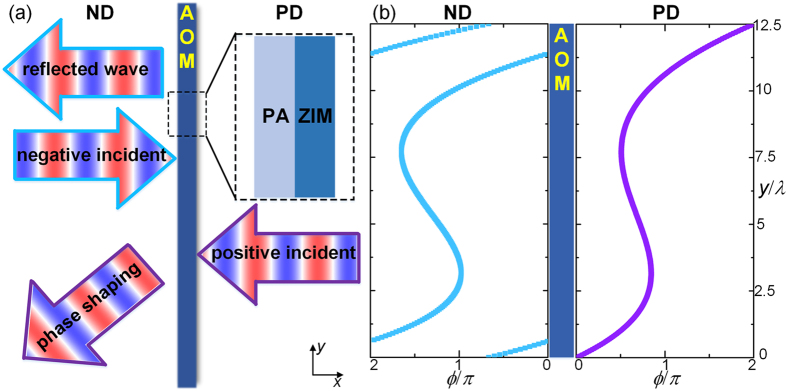
(**a**) Schematic of the proposed AOM design which is a combination of PA and ZIM as shown in the inset. (**b**) Schematic of the asymmetric phase manipulation of the AOM in PD and ND cases. Inset: a zoom-in of a part of the AOM.

The schematic diagram of the AOM is shown in Fig. 1(a), which is characterized by a subwavelength, planar configuration and the special wave-steering functionality that only works for incident wave from one particular side. The proposed AOM is a combination of acoustic PA and ZIM as shown in the inset of Fig. 1(a). In the current study, we define the propagating directions of the wave normally incident on the ZIM and PA as the positive direction (PD) and negative direction (ND) respectively. Only the acoustic plane wave incident from PD is permitted to transmit through the AOM and makes up the desired wavefront profile, while the wave propagating from ND is prohibited, as represented by the purple and blue arrows in Fig. 1(a), respectively. The PA offers a great deal of freedom and high efficiency in modulating the phase of transmitted wave flexibly that is, however, entirely symmetric in PD and ND. The extremely large phase velocity provided by the ZIM, on the other hand, leads to the near-zero critical angle and intrinsically high selectivity of the ZIM^27^. These result in two important features that are crucial for the effectiveness of the AOM: the high phase selectivity that only allows incident wave with uniform phase distribution in the transverse direction to penetrate while blocking the wave with non-uniform phase distribution, and the phase preservation capability that maintains the propagating phase of incident wave unchanged when passing through the planar ZIM and causes the transmitted wave and incident wave to share exactly the same phase profile. For the proposed AOM composed of the two components possessing their respective important features mentioned above, it can therefore be theoretically predicted that the AOM would behave differently in steering the phase of the incident wave in opposite directions, as will be proven later. This should be understood as the underlying mechanism responsible for the realization of one-way wavefront manipulation, which is inherently different from the mechanisms of the existing metasurface designs that provide symmetric phase responses only. This asymmetric phase manipulation ability of the AOM is well performed in Fig. 1(b). When the acoustic plane wave normally incident along the PD, the wave will be permitted to transmit through the ZIM due to the uniform phase profile which can be preserved until the wave leaves this part. Then the interaction with the PA will freely steer the phase to construct the desired phase profile 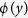, as represented by the purple dots in Fig. 1(b). In the ND, contrarily, the uniform phase profile of the incident wave has been disturbed and appended with phase 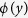 by PA before reaching the ZIM, leading to the blockage and reflection of the incident wave. The reflective wave in the output plane of the metasurface will be appended with phase 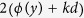 as indicated by the blue dots in Fig. 1(b), where *d* is the thickness of the PA in the propagation direction. As a consequence, a unidirectional transmission of acoustic waves is expected in the proposed system, and the wavefront of the transmitted wave in the PD can be arbitrarily engineered by designing different 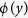 shown in Fig. 1(b).

**Figure 2:**
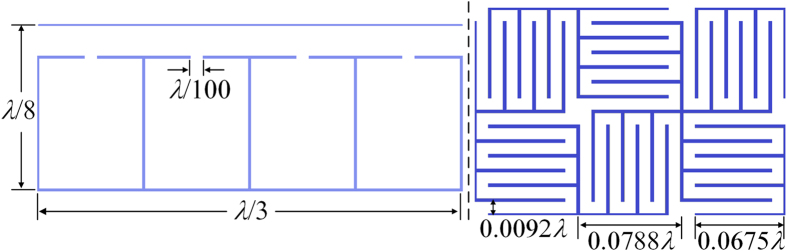
The hybrid metastructure (left part) and labyrinthine structure (right part) employed as the PA and ZIM in a particular implementation of the AOM.

In practice, the PA and ZIM, the two key components characterized by flexible phase control and extremely large phase velocity respectively, need to be implemented by real acoustical materials. Next we will demonstrate a practical implementation of the proposed scheme of AOM for airborne sound, as shown in Fig. 2. In this study we use three layers of labyrinthine structure designed by coiling up space and the hybrid metastructure comprising four Helmholtz cavities and a straight pipe as the ZIM and PA, respectively. In these two employed structures, the walls are regarded as rigid with a thickness of 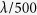, where *λ* is the sound wavelength. Other geometric parameters are shown in Fig. 2. Through adjusting the height of Helmholtz cavities one can manipulate the phase of the transmitted wave in the 2π range freely with almost-unity transmission^16^. And it has also been proven that the labyrinthine metastructure is able to behave as a ZIM by delicately adjusting its geometric paramters^27^,^29^. As a result, this implementation of AOM has a total thickness as thin as 0.57*λ* and a completely planar configuration. These important features would be particularly significant for practical applications due to the potential to minimize the size of devices and improve the compatibility to couple with the surroundings. Furthermore, it is worth pointing out that the general scheme proposed here for designing AOM are not exclusively restricted to these two particular configurations, but can also implemented by employing other candidates with tunable phase response and near-zero index to further improve the performance of the resulting device. This particular implementation composing of a hybrid metastructure and a labyrinthine structure for simplicity, has the optimal performance near the resonance frequency, where these two components are delicately designed to yield the near-zero index and the phase-modulating ability respectively. Beyond the designed working band, the performance of the implemented one-way metasurfaces will be affected, leading to reduction in the contrast factor. It is worth stressing however our proposed scheme of designing a one-way metasurface by coupling a phase array and a ZIM is general and should be irrelevant to the operating frequency. A broadband functionality can be achieved in practical implementations as long as specific kinds of metamaterials with broadband or non-dispersive near-zero indices are employed.

We have carried out a series of numerical simulations to verify our theoretical predictions as well as demonstrate the performance of the resulting AOM. As particular examples, three distinctive phenomena are chosen and will be exemplified in what follows: acoustic anomalous refraction, wave splitting and conversion of propagation wave to surface wave. The AOMs for these three examples have the same thickness 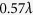, which are comprised of a layer of hybrid metastructure and three layers of labyrinthine structure.

**Figure 3:**
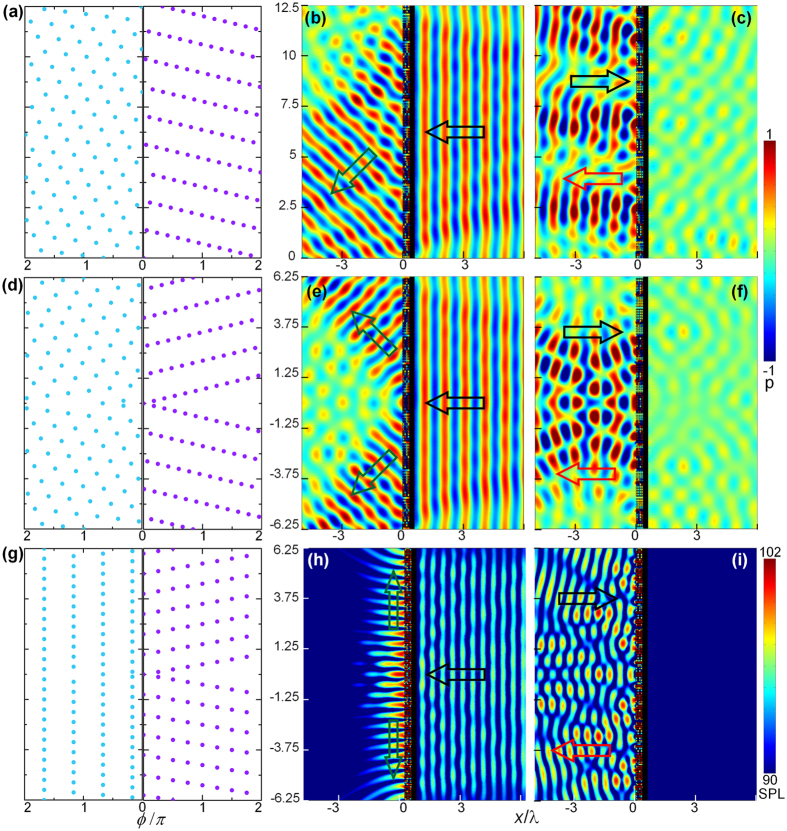
(**a**,**d**,**g**) The corresponding asymmetric phase profiles for realizing the anomalous refraction, wave splitter and the conversion of propagating wave into surface wave. (**b**,**c**,**e**,**f**) Acoustic pressure fields for the anomalous refraction and splitting acoustic wave in PD and ND cases, respectively. (**h**,**i**) Sound pressure levels for converting propagating wave into surface wave in PD and ND cases. The black arrows indicate the incident directions, the green and red arrows respectively denote the theoretical propagation direction for PD and ND cases.

Acoustic anomalous refraction is the phenomenon that the transmitted wave deviates from the incident direction when the wave impinges on a surface^31^. And the anomalous emergent angle, 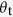, can be determined by designing the specific phase distribution 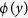 on the surface, which is governed by the generalized Snell’s law^31^:


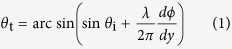


where *θ*_i_ is the incident angle, and the phase gradient term 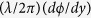 determines the anomalous emergent angle *θ*_i_. Here we choose a normally incident plane wave with *θ*_i_ being zero, and the emergent angle *θ*_t_ is selected to be 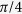. Figure 3(a) is the desired asymmetric phase profiles of the AOM in this situation, which can be realized by appropriately designing the geometric parameter of each unit cell of the hybrid metastructure mimicking the PA. The pressure patterns in PD and ND cases are shown respectively in Fig. 3(b,c), in which the black arrows denote the corresponding incident directions, the green arrow indicates the theoretical emergent angle and the red arrow is the reflection direction. As shown in Fig. 3(b), when the AOM is illuminated normally by a plane wave propagating in PD, full transmission and preserved phase distribution are permitted by the ZIM, and the designed wavefront profile of the transmitted wave is formed by the modulation effect of the PA. Excellent agreement can be observed between the theoretical prediction of the anomalous transmitted direction (green arrow) and the simulation result. On the contrary, the phase of the incident plane wave in the ND will be disturbed and attached with an additional phase by the PA first. Therefore, the ND incident wave is reflected back as shown in Fig. 3(c). For quantitatively evaluating the discrepancy between the transmissions in PD and ND case, a parameter of contrastive factor, defined as 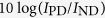 with 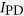 (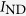) being the acoustic intensity in PD (ND) case, is introduced. The acoustic intensities are calculated by integrating the intensity in the corresponding surfaces perpendicular to the propagation direction. The numerical results reveals that the contrastive factor in this situation reaches 12.5 dB, which verifies the high efficiency of the distinct unidirectional property of the proposed AOM.

Then we present the realization of splitting acoustic beam based on the proposed scheme, with the plane wave being confined and guided to two symmetric directions with respect to the incident direction^32^,^33^ in the PD situation and reflected back in the ND situation. The asymmetric phase profiles of the AOM in PD and ND cases are shown in Fig. 3(d), and the corresponding acoustic pressure fields in PD and ND cases are displayed in Fig. 3(e,f), in which the black, green and red arrows indicate the incident, theoretical transmitted and reflected directions respectively. Acoustic plane wave incident from PD is able to pass through and shifted into two symmetric beams as expected. However, in ND case the incident wave is prohibited to penetrate and is subject to a total reflection. In such a case, a contrastive factor of 13.2 dB is obtained. The asymmetric splitting ability of the AOM can be applied in constructing one-way beam-splitting prisms, which may have wide applications in practice.

Finally, we consider the conversion of acoustic propagating wave into surface wave with the AOM, which is the wave bounded at the surface and evanescent on the transmitted side^34^. The transmitted wave with the angle 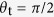 can be obtained according to Eq. (1), and the desired phase profile is illustrated in Fig. 3(g). Similarly, the PD incident wave is allowed to transmit through the ZIM and converted to the surface wave, while the ND incident wave is prohibited. The corresponding sound pressure levels (SPLs) in PD and ND cases are shown in Fig. 3(h,i), respectively. The results clearly demonstrate that the acoustic field is obviously enhanced on the surface and attenuates quickly away from the interface, revealing the nearly perfect conversion of the propagating wave into the surface mode. Physically, in the PD case, the discrete phase shifts provided by the PA along the *y* direction provide extra momenta to compensate the momentum mismatch between the propagating wave and the surface wave on the metasurface, resulting in the high efficiency conversion. The asymmetric transmission property of the AOM is verified by the 12.1 dB contrast of the sound intensity between the opposite cases. The asymmetric conversion with the AOM could act as a bridge to link the propagation wave and surface wave, which would lead to both the theoretical interests and practical applications.

## Discussion

The design of an AOM has been proposed, which is capable of manipulating the wavefront of transmitted wave flexibly in only one direction but blocking the transmission in the opposite direction, and demonstrated a practical implementation by metamaterials via numerical simulations. This intriguing characteristics results from a distinct ability of asymmetric phase manipulation achieved by coupling PA with ZIM which are implemented by employing hybrid metastructures and labyrinthine units respectively. As a few examples, we have investigated the acoustic anomalous refraction, splitting wave and converting the propagating wave into surface wave to demonstrate the performances of the proposed AOM. The subwavelength thickness and planar configuration of the particular implementation would contribute to the practical applications. We need to emphasize that the proposed AOM has not broken the reciprocity principle and the time-reversal symmetry is still valid in this system, which limits the one-way transmission functionality to the normal incidence case. Due to the high selectivity of the ZIM, the oblique incident wave will be totally reflected from the surface as well. With the capability of realizing asymmetric transmission and wavefront manipulation simultaneously with high efficiency, the proposed AOM offers a framework in integrating the unidirectional propagation with versatile wave manipulation, which may find applications in a great variety of scenarios such as ultrasound imaging or therapy where special control of acoustic transmission is always highly desired.

## Method

Throughout the paper, the numerical simulations are performed by using finite element method based on COMSOL Multiphysics software. The background medium is air, the mass density and sound speed of it is 1.21 kg/m^3^ and 343 m/s. The material of the AOM in simulation is chosen to be acrylonitrile butadiene styrene (ABS) plastic, whose mass density and sound speed are 1200 kg/m^3^ and 2200 m/s, respectively. Perfectly matched layers (PMLs) are utilized to eliminate the reflected waves by the outer boundaries.

## Additional Information

**How to cite this article**: Jiang, X. *et al*. Acoustic one-way metasurfaces: Asymmetric Phase Modulation of Sound by Subwavelength Layer. *Sci. Rep.* **6**, 28023; doi: 10.1038/srep28023 (2016).
